# Impact of the COVID-19 Pandemic on Acute General Surgical Admissions in a District General Hospital in the United Kingdom: A Retrospective Cohort Study

**DOI:** 10.1155/2020/2975089

**Published:** 2020-08-12

**Authors:** Rory Callan, Nazrin Assaf, Katharine Bevan

**Affiliations:** Department of General Surgery, Bedford Hospital, Bedfordshire Hospitals NHS Foundation Trust, Bedford MK42 9DJ, UK

## Abstract

The COVID-19 pandemic of 2020 has greatly impacted healthcare systems and society more generally around the world. The management of patients infected with SARS-CoV-2 has primarily impacted emergency departments, medical teams, and intensive care units. However, the impact on health systems as a whole, including surgical specialties, has been wide ranging. We aimed to establish the impact of the COVID-19 pandemic and associated lockdown on the number and characteristics of general surgical patients reviewed and/or admitted by the surgical team within a district general hospital. We performed a retrospective cohort analysis of patients admitted in the 2-week period from start of the lockdown (Monday 23rd March 2020 to 5th April 2020), and the same period 1 year earlier (Monday 25th March 2019 to 7th April 2019). Number of patients reviewed and admitted were compared between the two cohorts. Data including diagnosis, operation/procedural interventions, and length of stay were analysed. The overall number of patients reviewed and admitted by the surgical team was substantially lower during the period of lockdown (61 vs 126). Of the patients seen during lockdown, a smaller proportion were admitted to hospital after initial surgical review (59% vs 77%, *p* < 0.05). Interventional/operative procedures were performed in a similar proportion of patients in both cohorts (31%). Our data show that there has been a substantial reduction in the number of patients being referred to and admitted by the general surgical team at our centre during the COVID-19 pandemic. Explanations for this include reduced attendance due to risk perception of the patients, the impact of lockdown messages and advice regarding self-isolation, as well as an increased threshold for patient admission during the COVID-19 pandemic. Key learning points include the possible benefits of a reduction in admission to hospital of patients with nonurgent conditions.

## 1. Introduction

The coronavirus-disease 2019 (COVID-19) pandemic of 2020 has caused huge disruption in the functioning of healthcare systems across the world [1]. The UK health system, primarily made up of the National Health Service (NHS), has seen a drastic change in how services are provided, and the pressures are expected to increase over the course of the pandemic [2]. Hospitals have been expected to come under increasing strain as cases increase and intensive care beds are filled. Social distancing measures that aim to “flatten the curve” and reduce peak incidence of infection have been put in place by governments across the world, including the UK government [3].

On the 23rd of March 2020, the UK Prime Minister Boris Johnson announced the following three measures aimed at reducing spread of the virus: requiring people to stay at home, except for very limited purposes, closing certain businesses and venues, and stopping all gatherings of more than two people in public.

The impact of these measures, and the COVID-19 pandemic more generally on hospitals and patients with conditions other than coronavirus, has been discussed throughout the pandemic. Data released by NHS England have shown that there has been a reduction of 29.4% in the number of A&E attendances in the month of March 2020, compared to the same period last year [4]. There have been concerns raised that patients with conditions unrelated to COVID-19 may be delaying presenting to hospital, increasing risk of morbidity and mortality [5].

Many changes in surgical practice have been enacted during the COVID-19 pandemic. NHS England advised all trusts on the 17th of March 2020 to cancel all nonurgent surgeries for at least three months, to free up capacity for patients admitted with COVID-19 [6]. The Royal Colleges have released guidelines on the management of acute surgical conditions, which include advice to manage conditions conservatively when possible, including acute cholecystitis and acute appendicitis [7].

Our aim was to investigate the impact of the COVID-19 pandemic and associated lockdown on attendances and admissions of general surgical patients within a district general hospital in the United Kingdom. We also set out to establish whether there were positive learning points to be taken from changes in practice during the COVID-19 pandemic, including the impact of a possible increase in the threshold for patient admission.

## 2. Materials and Methods

### 2.1. Study Setting

The study was conducted at Bedford Hospital, a 400-bed district general hospital. It has yearly A&E attendances of approximately 76,000 patients, with 55,000 admissions [8]. As well as general surgery, it provides vascular, urological, ENT, and plastic surgery services. This study focused on general surgical patients as these make up the majority of daily attendances and admissions. There is a general surgical registrar/middle grade on site 24 hours, 7 days a week, as well as a FY2/house officer and FY1.

### 2.2. Study Design and Procedure

We conducted a retrospective review of notes of patients reviewed by the emergency general surgical team at a district general hospital. Two periods were examined: the 2-week period after the announcement of the UK government mandated lockdown, from the 23rd March 2020 to 5th April 2020, and the same period 1 year earlier—25th March 2019 to 7th April 2019.

All general surgical patients reviewed and/or admitted by the on-call team were included, taken from the daily handover list. All patients with a nongeneral surgical pathology (e.g., urological or vascular issue) were excluded. Data were collected on patient age, date of admission and discharge, most senior doctor to review patient prior to discharge, length of stay, operative and interventional procedure, and mortality/morbidity, as well as diagnosis on discharge. We also collected 30-day follow-up data for the cohort discharged during the COVID-19 pandemic, to establish any adverse events and readmissions in this patient group.

### 2.3. Statistical Analysis

The proportion of patients who were reviewed by the surgical team and admitted to hospital and who underwent interventional procedures and their length of stay were analysed for statistical significance. Level of statistical significance was established using the chi-square test/Fisher's exact test for proportion of patients admitted to hospital and surgical intervention, and the Student standard *T* test for length of stay.

## 3. Results and Discussion

### 3.1. Results

The total number of patients reviewed and/or admitted by the surgical team was substantially lower in the 2020 cohort than the 2019 cohort (61 vs 126 patients), a 52% reduction in patients presenting to the general surgical team.

**Figure 1:**
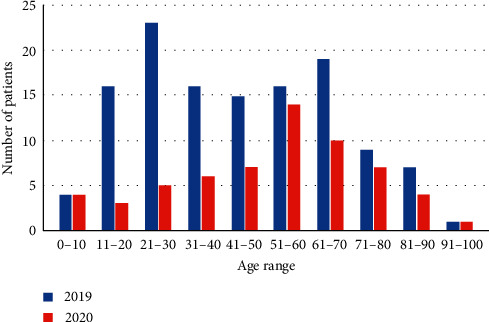
The number of patients reviewed by the surgical team in 2019/2020 cohorts by age.

The age distribution of each cohort was analysed (Figure 1).

As can be seen, there was a reduction of most age ranges presenting to the surgical team in 2020 compared to 2019. However, this decrease was greater in the younger patient populations, with a 69% decrease in patients aged 40 and under compared to a 30.1% reduction in patients aged 41 and over.

**Figure 2:**
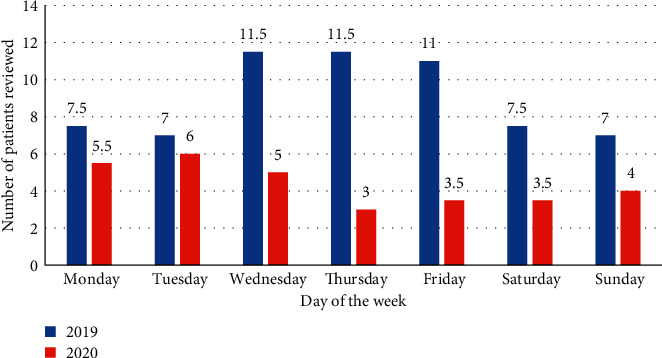
The average (mean) number of patients reviewed each day of the week by the surgical team, in the 2-week period in 2019 and 2020.

The average number of patients reviewed per day in each period was analysed (Figure 2).

These data show that there was a consistent reduction in patients reviewed on every day over the 2-week period in 2020 compared to 2019.

**Figure 3:**
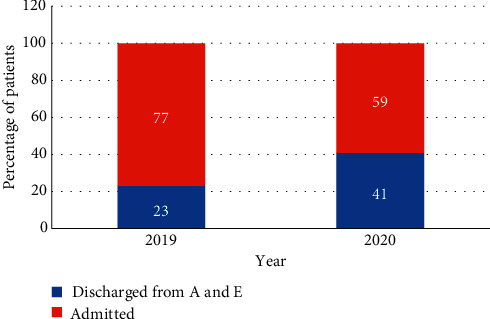
The proportion of patients admitted to hospital after initial review was significantly higher in the 2019 cohort.

We then reviewed the proportion of patients that were admitted to hospital in each cohort. Of the patients reviewed by the surgical team, a significantly lower proportion of patients were admitted in 2020 (59% in 2020 vs 77% in 2019, *p* < 0.05) (Figure 3).

Of the patients discharged from the emergency department in the 2020 cohort, the majority, for which data were available, were reviewed by a middle grade/SpR surgeon before discharge (81%, *n* = 18). Three patients were reviewed by an FY2/SHO (14%), and one patient was reviewed by a consultant prior to discharge. This is in keeping with usual practice within the department, where the majority of patients are reviewed prior to discharge by the on-call middle grade/SpR.

**Figure 4:**
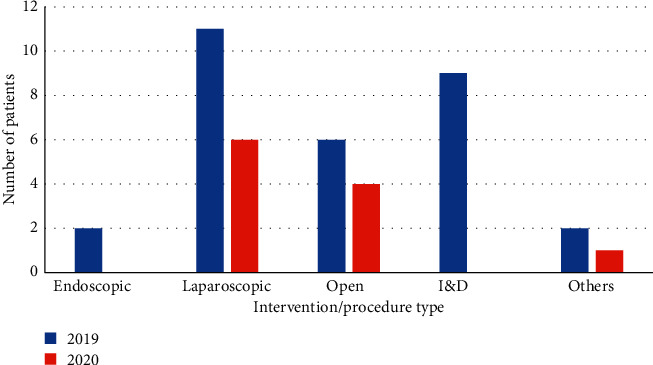
Number of patients undergoing interventional or diagnostic procedures in 2019/2020 cohorts.

Diagnostic, interventional, and/or operative procedures were performed in a similar proportion of patients in both cohorts (31% in both cohorts). However, the type of procedure varied—no endoscopic procedures were carried out on patients admitted under the surgical team in the 2020 cohort, and no patients underwent incision and drainage procedures (see Figure 4). This is in keeping with guidance from the British Society of Gastroenterologists to avoid all but emergency endoscopic cases [9], with a higher threshold for endoscopic intervention during the COVID-19 pandemic. The rate of open and laparoscopic procedures was higher in the 2020 cohort (Table 1), possibly indicating a reduction in admission of patients not requiring operative intervention—however, these differences did not reach statistical significance.

We examined the diagnosis on discharge for all patients reviewed and/or admitted during the two periods. In both, the most common diagnoses in admitted patients were nonspecific abdominal pain, appendicitis, bowel obstruction, and cholecystitis (see Table 2). However, the proportion of patients admitted with nonspecific abdominal pain was lower in the 2020 cohort (33% vs 67%, *p* < 0.05), perhaps reflecting a higher threshold for admission during the coronavirus pandemic. This was also true in patients presenting with biliary colic, breast infections, and gastritis, where there was an increase in proportion of patients discharged in 2020.

**Figure 5:**
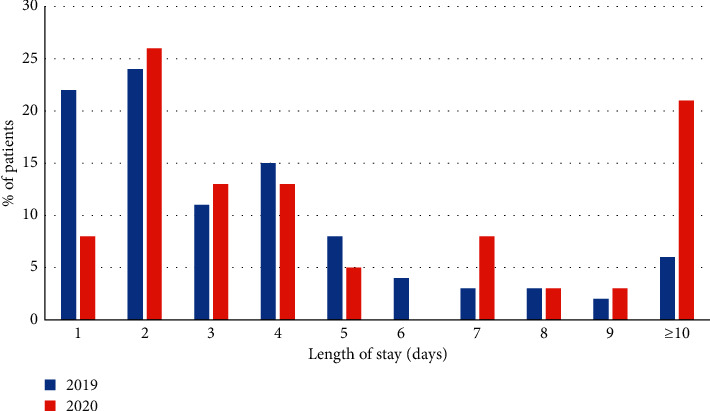
Length of stay data, with percentage of admitted patients in each cohort.

Length of stay data showed an increase in median (3.5 days vs 3 days) and mean (5.7 vs 3.2 days) length of stay in the 2020 cohort (see Figure 5). This difference was statistically significant, with a *p* value = 0.0133 (95% CI: −3.43 to −0.41 days). The number of patients admitted for 1 day in the 2019 cohort was 22%, vs 8% in 2020. A substantially higher proportion of patients were admitted for 10 days or more in the 2020 cohort (21% vs 6%). These data may reflect an increased severity of illness in the patients presenting in 2020, and reduction in the number of patients admitted with diagnoses of nonspecific abdominal pain, biliary colic, and other less acute general surgical conditions. It may also reflect an increase in conservative management of conditions such as appendicitis and cholecystitis, which has been found in previous studies to increase length of stay [10].

We followed up the patients who were not admitted to hospital in the 2020 cohort, to determine the number of re-attendances within 30 days, and also reviewed interventions that were carried out during this second presentation. Of the 25 patients in this group, 22 patients (88%) did not re-attend hospital within 30 days. Three patients that re-attended were admitted to hospital, with one patient undergoing an intervention (laparoscopic appendicectomy). These results indicate that these patients were safely discharged, with no documented adverse events resulting from discharge from A&E.

**Figure 6:**
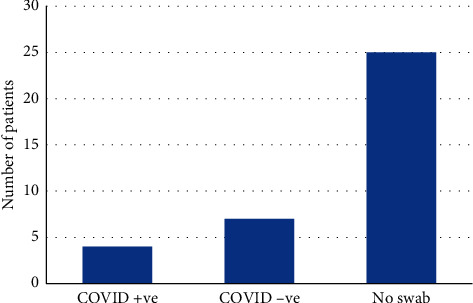
Patients in 2020 cohort who were swabbed/not swabbed for SARS-CoV-2, with 4 patients testing positive, 7 testing negative, and 25 not receiving swabs.

Finally, we examined the mortality data within the two cohorts, as well as SARS-CoV-2 status in the 2020 cohort. The 2019 cohort had 1 mortality (mortality rate of 1.03%). The 2020 cohort had 4 deaths (mortality rate of 11.1%). One of these mortalities was related to COVID-19—this patient was diagnosed with COVID-19 12 days after initial admission, indicating that they potentially caught the virus while admitted, highlighting the importance of minimising unnecessary admissions during the pandemic. There were 4 patients tested and found to be positive for SARS-CoV-2 in the 2020 cohort (Figure 6). Although these mortality figures are not statistically significant, they do highlight the possible risk to surgical patients of admission during the COVID-19 crisis and mortality associated with SARS-CoV-2 infection during admission.

## 4. Discussion

Our data show that there has been a substantial reduction in the number of patients being referred to and admitted by the general surgical team at our centre during the COVID-19 pandemic. This is in keeping with the findings in NHS England data, showing a substantial reduction in A&E attendances in general.

The greater length of stay may indicate an increase in severity of cases that are presenting to the general surgical team. An increase in the average length of stay and specifically the number of patients staying for 10 or more days was seen in the patients admitted during the COVID-19 pandemic. With an increased focus on discharging medically fit patients during the COVID-19 pandemic, this is unlikely to be solely due to discharges being delayed due to social issues.

There is a risk that patients with serious surgical pathology have delayed presentation to A&E or other healthcare professionals due to the messaging surrounding the lockdown, and concern about the risk of becoming infected with SARS-CoV-2 if admitted to hospital. Recent data from the United Kingdom have shown an increase in mortality above that expected with just deaths related to COVID-19 taken into account [11].

To ensure that going forward there is public confidence in the safety of attending hospital, there is going to need to be significant work on the development of “green” pathways. These aim to reduce the risk to patients presenting either to A&E or as an outpatient for clinic appointments or theatre. Our centre has developed separate pathways for elective patients, with negative swabs and 2 weeks of self-isolation necessary prior to attendance for elective cases. The use of video-conferencing and telephone consultations has increased significantly during the pandemic—this will likely need to continue into the future and may lead to improved patient experience, with fewer journeys to hospital, and increased capacity for outpatient appointments.

There are other positive learning points that can be taken from changes in acute surgical practice during the COVID-19 pandemic. Increasing the threshold for patient admission during the COVID-19 pandemic did not lead to any documented adverse outcomes in our patient cohort. The proportion of patients admitted with conditions that can often be managed safely on an outpatient basis, including biliary colic, gastritis, and nonspecific abdominal pain decreased during the COVID-19 pandemic. Use of “hot clinics” in which patients with nonurgent conditions can be managed as an outpatient with timely imaging and treatment, can reduce the proportion of patients admitted and is a way in which our findings during this pandemic might be used to alter practice into the future.

This has implications for resources—the cost of an overnight stay was estimated in 2015 to cost £400 [12]. By reducing the proportion of patients being admitted for 1 day to levels seen in the 2020 pandemic, a total of £5200 could have been saved in just the 2-week period in 2019 included in the study. More evidence needs to be gained on the safety of this approach. However, our data showed no adverse events from this increased threshold for admission. There are potential benefits for patients, including reduced time in hospital, and the surgical team including possible cost savings and the ability to focus on unwell patients.

As the incidence of COVID-19 begins to decrease and we see a subsequent easing of lockdown measures, there are still likely to be longstanding changes in how surgical teams function. This makes it all the more crucial to learn lessons from the pandemic and to continue to apply these in the coming years.

## 5. Conclusions

There has been a large reduction in patients presenting with emergency general surgical conditions to a district general hospital in the United Kingdom during the COVID-19 pandemic and associated lockdown. A significantly lower proportion of patients reviewed by the general surgical team were admitted in the 2020 cohort (59% vs 77%). Length of stay was significantly longer during the COVID-19 lockdown, with 21% of patients admitted for 10 or more days (6% in 2019). These findings should prompt a discussion as to degree of excess morbidity and mortality suffered by general surgical patients during the COVID-19 pandemic. However, there are also some key learning points—a reduction in patients admitted with nonurgent conditions did not lead to any observed adverse outcomes in the 2020 patient cohort, with positive implications for resource allocation.

## Figures and Tables

**Table 1:** Details of the procedures carried out in each cohort, with totals for each subgroup of procedure and individual procedure type, and the percentage of patients undergoing each procedure, with level of statistical significance shown in brackets.

Procedure type	2019		2020	
	No. of patients	% of total admitted patients undergoing intervention (%)	No. of patients	% of total admitted patients undergoing intervention (%)
*Endoscopic procedures*	**2**	2	0	0 (*p*=1)
ERCP	1		0	
Flexi Sig	1		0	

*Laparoscopic procedures*	**11**	11	6	17 (*p*=0.4)
Appendicectomy	10		3	
Diagnostic laparoscopy	1		1	
Adhesiolysis	0		1	
Small bowel resection and loop ileostomy	0		1	

*Open procedures*	**6**	6	4	11 (*p*=0.46)
Right hemicolectomy	2		0	
Inguinal hernia repair	1		1	
Appendicectomy	1		0	
Ileostomy reversal	1		0	
Laparotomy for bleeding duodenal ulcer	1		0	
Diaphragmatic hernia repair	0		1	
Hartmann's procedure	0		1	
Femoral hernia repair and small bowel resection	0		1	

*Incision and drainage*	**9**	9	0	0 (*p*=0.11)
Perianal abscess	2		0	
Pilonidal abscess	3		0	
Other abscess	3		0	
Breast abscess	1		0	

*Others*	**2**	2	1	3 (*p*=1)
Chest drain	1		1	
Manual evacuation	1		0	

*Total procedures* (*no. of admitted patients*)	**30** (97)	31	11 (36)	31

**Table 2:** The diagnosis of patients reviewed by the surgical team.

Diagnosis	2019		2020		% change in % patients discharged (2020 vs. 2019) (%)
	Admitted (no. of patients)	Discharged (no. of patients)	Admitted (no. of patients)	Discharged (no. of patients)	

Nonspecific abdominal pain	18	9	4	9	+36
Appendicitis	11	0	4	1	+20
Bowel obstruction	8	0	5	0	0
Cholecystitis	6	0	4	0	0
Abscess (including perianal, pilonidal, others)	8	2	0	0	NA
Breast infection (abscess/mastitis)	3	3	0	2	+50
Biliary colic	3	2	0	2	+60
Post-op complication	3	1	1	2	+42
Pancreatitis	3	0	3	0	0
Diverticulitis	3	0	3	0	0
Constipation	2	2	1	1	0
Gastritis	2	3	0	1	+40
PR bleed	2	1	1	1	+17
Ovarian cyst	4	0	0	0	NA
Colitis	1	0	1	1	+50
Chest trauma	1	0	1	1	+50
Hernia	1	0	0	2	+100
Mesenteric adenitis	0	1	1	1	−50
GI perforation	1	0	1	0	0
Adhesional pain	2	0	0	0	NA
Volvulus (sigmoid/caecal)	2	0	0	0	NA
Gastroenteritis	1	1	0	0	NA
Progression of malignancy	1	0	1	0	0
Thrombosed pile	0	1	0	1	0
Colorectal cancer	0	0	2	0	0
Others	11	3	3	0	NA

Total number of patients that were admitted and discharged are included, as well as the change in the % of patients discharged. All diagnoses seen in only 1 patient are included under “Others.”

## Data Availability

The data used to support the findings of this study are available from the corresponding author upon request.
